# Burn Injury Treatment Outcomes and Associated Factors Among Patients Admitted to a Tertiary Hospital Burn Unit in Southern Ethiopia: A Facility‐Based Cross‐Sectional Study

**DOI:** 10.1002/hsr2.72679

**Published:** 2026-06-18

**Authors:** Sewnet Ejigu, Tesfaw Alemu Legas, Samson Kastro Dake, Natnael Sima, Samuel Minalkew, Mulugeta Edao Shate, Wondimagegn Genaneh Shiferaw

**Affiliations:** ^1^ School of Medicine Wolaita Sodo University Sodo City Ethiopia; ^2^ Department of Midwifery Wolaita Sodo University Sodo City Ethiopia; ^3^ School of Public Health Wolaita Sodo University Sodo City Ethiopia; ^4^ Department of Nursing Hawassa University Hawassa Ethiopia; ^5^ Department of Emergency and Critical Care Nursing Wolaita Sodo University Sodo City Ethiopia

**Keywords:** associated factors, burn injury, burn outcome and Ethiopia

## Abstract

**Background and Aims:**

Burn injuries, caused by heat, radiation, electricity, friction, or chemical exposure, result in significant damage to the skin and underlying tissues, representing a leading cause of morbidity and mortality, particularly in developing countries. Despite their substantial impact on health outcomes, evidence regarding burn injury outcomes and contributing factors remains limited in the study area. This study aimed to assess treatment outcomes and associated factors among burn patients admitted to the burn ward of the University Comprehensive Specialized Hospital between November 2020 and November 2024.

**Methods:**

This facility‐based cross‐sectional study included 271 burn patients selected using consecutive sampling. Data were extracted from medical records using structured checklists via Kobo Collect, exported to SPSS version 26 for analysis. Multivariable analysis identified factors significantly associated with burn outcomes at *p*‐value < 0.05.

**Results:**

The prevalence of discharge with complications was 17.7% (95% confidence interval [CI]: 13.0%–22.0%). Independent predictors included age ≥ 60 years (adjusted odds ratio [AOR] = 4.40; 95% CI: 1.01–19.11), pre‐hospital interventions (AOR = 2.34; 95% CI: 1.15–4.98), absence of fluid and electrolyte replacement (AOR = 3.06; 95% CI: 1.39–6.70), and burn surface area ≥ 30% (AOR = 5.20; 95% CI: 1.53–17.64).

**Conclusion:**

Advanced age, inappropriate pre‐hospital interventions, insufficient fluid resuscitation, and extensive burns (≥ 30% total body surface area [TBSA]) were identified as key independent determinants of discharge with complications. Targeted strategies including age‐tailored management, community burn first aid education, and adherence to fluid resuscitation protocols are essential to reduce burn‐related complications and improve outcomes in resource‐limited settings.

**Abbreviations**
AaBET: Addis Ababa burn, emergency and trauma
CI: confidence interval
DALY: disability adjusted life year
LMICS: low and middle‐income countries
SPSS: statistical package for social sciences
TBSA: total body surface area
UCSH: University Comprehensive Specialized Hospital
WHO: World Health Organization

## 1 Introduction

Burn injuries, as defined by the World Health Organization (WHO), are the result of skin and underlying tissue damage from exposure to fire, scalding liquids, electrical currents, chemicals, or radiation. These injuries require intensive medical resources, involving prolonged hospitalization, complex wound care, and extensive rehabilitation. Burns are one of the most costly traumatic injuries to treat globally due to the need for long‐term recovery interventions, such as surgeries and scar management [1, 2, 3].

Burn injuries can be classified based on depth, etiology, and the percentage of total body surface area (TBSA) affected. Etiologically, burns are categorized into thermal (scalds, dry heat, and contact burns), electrical, chemical, and radiation‐induced injuries [3, 4]. Depending on the depth, burns are further divided into superficial (first‐degree), partial thickness (second‐degree), full thickness (third‐degree), and deeper injuries involving muscle, bone, or nerves (fourth‐degree). Injuries covering less than 10% TBSA are often considered minor, while those affecting over 20% TBSA are categorized as major burn injuries [5, 6].

Burns rank as the fourth most common cause of trauma globally, following road traffic accidents, falls, and interpersonal violence [4]. WHO estimates that around 265,000 deaths occur annually due to burn injuries, with approximately 90% of these fatalities occurring in low‐ and middle‐income countries (LMICs) where resources are constrained and healthcare infrastructure is limited [3, 7]. In sub‐Saharan Africa, burn injuries are especially concerning, with pediatric burn mortality rates reaching as high as 27.8%, underlining the critical need for targeted prevention strategies and improved treatment access in regions with limited healthcare infrastructure [8, 9].

Burn injuries remain a critical health challenge worldwide. The WHO reported in 2018 that around 11 million burn injuries occur each year, with 180,000 resulting in death. And it's also a leading cause of disability, contributing to over 8 million disability‐adjusted life‐years (DALYs) annually [10, 11]. Severe burns provoke physiological reactions akin to those seen in major trauma, such as venous thromboembolism, which further complicates recovery, increases morbidity, and heightens the risk of mortality [12]. Non‐fatal burn injuries carry profound physical, psychological, and social consequences. Survivors often experience lasting physical disabilities, scarring, and mental health challenges, as well as social stigma and economic hardship, due to prolonged recovery needs and reduced physical function [13, 14].

Although burn injury rates have decreased in high‐income countries, LMICs, where 90% of all burns occur, still experience high prevalence, morbidity, and mortality. These injuries impose substantial financial burdens due to high treatment costs, extensive rehabilitation, and lengthy hospital stays. In Ghana, the average cost of burn treatment is USD 3897.58 [11, 15, 16]. Burns impose a considerable financial burden on healthcare systems as well; for example, the average hospitalization cost per burn patient is approximately $32,360 in Canada, while in Nepal, treatment expenses can surpass twice the national average per capita income [17].

Proposed solutions include establishing specialized burn units, training healthcare providers in burn management, and promoting community‐based prevention programs to reduce burn incidence. Additionally, WHO advocates for affordable, locally adapted interventions, such as low‐cost wound care techniques and targeted education on fire safety in high‐risk communities [18, 19, 20].

In LMICs, burns are not only a leading cause of mortality but also impose a significant burden on health systems due to high morbidity rates. Populations like children and the elderly are particularly vulnerable to severe outcomes, experiencing long hospital stays, social isolation, and a lower quality of life due to burn injuries [21, 22]. In Ethiopia, where burn care facilities are scarce, understanding the magnitude and impact of burns is essential to developing effective prevention measures and consistent treatment protocols, especially in regions facing limited resources and infrastructural challenges [23].

In Sub‐Saharan Africa, the burden of burn injuries is especially high. Studies show that the mortality rate among burn patients in this region ranges from 6% in Sudan [24] to as high as 17% in other areas [16]. Vulnerable populations, such as children and lower‐income individuals, face the greatest risk, often lacking access to specialized care. In Ethiopia, limited data are available on burn outcomes and associated factors impacting patient prognosis. However, studies at AaBET Hospital revealed a complication rate of 14.9% and a mortality rate of 6.6% among burn patients, with the highest complication rates among adult males [23].

Previous studies have reported high incidence and severe outcomes of burn injuries in LMICs, where the combination of limited healthcare infrastructure and socioeconomic challenges exacerbates patient outcomes, with mortality rates significantly higher in LMICs due to inadequate burn care facilities, lack of trained personnel, and scarcity of resources for long‐term care and rehabilitation [18, 25].

In Ethiopia, the implementation of effective burn prevention and control strategies is hampered by limited health infrastructure, highlighting the urgent need to adapt and optimize interventions in resource‐poor settings. Despite the substantial public health burden of burn injuries, data on burn outcomes and associated risk factors remain scarce at the regional level. This paucity of evidence impedes the ability of healthcare providers to design context‐specific treatment protocols and prevention measures. This study therefore aims to address this gap by examining burn treatment outcomes and the factors that influence them at the University Comprehensive Specialized Hospital (UCSH), with the ultimate goal of providing clinicians and policymakers with locally relevant, evidence‐based information to improve burn care and reduce the physical, social, and economic consequences of burn injuries in South Ethiopia.

## 2 Methods

### 2.1 Study Area

This study was conducted at the UCSH, located in Wolaita Sodo Town, Southern Ethiopia, approximately 328 km south of Addis Ababa. The hospital serves as a teaching and referral institution with 350 beds, including 52 surgical beds, and caters to a catchment population of over 3 million people [26, 27, 28, 29]. The hospital maintains a single specialized burn unit that provides care for a diverse patient population, encompassing pediatric, adult, and female patients presenting with a wide spectrum of burn injuries. The unit is staffed by more than 20 healthcare professionals, including surgery residents, and delivers comprehensive multidisciplinary management spanning acute resuscitation through to ongoing rehabilitation.

### 2.2 Study Design and Period

A retrospective chart review was employed from November 1–30, 2024.

#### 2.2.1 Source and Study Population

##### 2.2.1.1 Source Population

All burn patients admitted to the Hospital from November 2020 to 2024.

#### 2.2.2 Study Population

The study population included all burn patients admitted to the surgical burn ward who were selected as study subjects during the study period and meeting the inclusion criteria.

### 2.3 Inclusion and Criteria

#### 2.3.1 Inclusion Criteria

Medical records of all patients admitted with burn injuries to the burn ward at the UCSH during the study period were eligible for inclusion. Charts were included if they contained complete and consistent documentation of socio‐demographic characteristics, clinical presentation, burn‐related characteristics, treatment interventions, and discharge outcomes.

Patients across all age groups‐ pediatric, adult, and elderly, were included to provide a comprehensive assessment of burn outcomes across the hospital population. This inclusive approach enabled evaluation of common predictors and system‐level factors, such as pre‐hospital care, fluid resuscitation, and burn surface area (BSA), which influence outcomes irrespective of age. Age was treated as an independent variable in the multivariable analysis to account for physiological differences and differential risk profiles, while the inclusion of all age groups enhanced statistical power to detect significant associations with treatment outcomes.

#### 2.3.2 Exclusion Criteria

Medical records were excluded if they had substantial missing or inconsistent information on key study variables, including burn severity, management, or treatment outcome. Charts of patients who were transferred to another health facility before a definitive outcome was documented were excluded. Records of patients who left against medical advice, absconded, or had undocumented discharge status were also excluded from the analysis.

### 2.4 Sample Size Determination

The sample size for the first objective was calculated using the single population proportion formula, based on a prevalence of 21.5% for patients discharged with complications, derived from a study on burn injury outcomes and associated factors conducted at AaBET Hospital [12]. Assuming a 95% confidence interval and a margin of error of 5%, the calculated sample size was 260. Adding a 10% non‐response rate yielded a final sample size of 286.

The formula applied was as follows:

$$n=(Z^{2}\times p(1-p))/d^{2}$$

Where *n* is the desired sample size, *Zα*/2 is the critical value at 95% confidence interval (1.96), *p* is the proportion of burn injury patients discharged with complications (0.215), and *d* is the margin of error (0.05).

The sample size for the second objective, identifying associated factors, was determined using the double population proportion formula, calculated with Epi Info version 7.2.1. Considering age and BSA as key variables, with assumptions of 80% statistical power, a 95% confidence interval, and an exposed‐to‐unexposed ratio of 1:1, the calculated sample size was 76. As the sample size derived from the first objective was larger, it was adopted as the final sample size of 286 for the study. The final sample size becomes 286 (See Table 1 for more information).

**Table 1:** Sample size calculation using associated factors to assess the management outcome of burn injury & its associated factors at CSH.

Variables	Assumption	Proportion	AOR, *p* value	Sample size	Reference
Age > 60 yrs.	CI = 95, P = 80%, un/e = 1	P1 = 0.25, P2 = 0.0096	2.21 [1.32–3.69], 0.037	76	[23]
Percent of TBSA > 30%	CI = 95, P = 80%, un/e = 1	P1 = 0.55, P2 = 0.0171	8.72 [1.32–57.33], 0.012	28	[23]

### 2.5 Sampling Technique and Data Collection Tool

A total of 295 patient records were identified from admissions to the burn ward between November 2020 and November 2024. Data were collected retrospectively during November 2024. Of the 295 identified records, 286 were selected using consecutive sampling. Following review for completeness, 15 charts were excluded due to incomplete documentation of essential variables, leaving 271 charts for final analysis. Data were extracted using a structured questionnaire developed following an extensive review of relevant literature from studies conducted worldwide. The questionnaire, prepared in English, was designed to capture detailed information on socio‐demographic characteristics, clinical factors, and treatment‐related variables.

### 2.6 Variables of the Study

#### 2.6.1 Dependent Variable

The outcome of burn injuries (discharged with complications vs. discharged without complications).

#### 2.6.2 Independent Variables

##### 2.6.2.1 Socio‐Demographic Factors

Age, sex, marital status, residence.

##### 2.6.2.2 Clinical Factors

Comorbidity, time to reach facility, cause of burn, type of burn, location of burn, TBSA%, degree of burn, duration of admission.

##### 2.6.2.3 Treatment‐Related Factors

Pre‐hospital intervention, intervention type, fluid resuscitation.

### 2.7 Operational Definitions

#### 2.7.1 Burn Patients

An individual who has sustained a burn injury requiring hospital admission and medical care.

#### 2.7.2 Burn Injury

Damage to the skin or underlying tissues resulting from thermal, chemical, electrical, or radiation exposure [30].

#### 2.7.3 Type of Burn Injury

Burn injuries are classified according to depth (superficial, deep partial‐thickness, and full‐thickness) and by the percentage of TBSA affected.

#### 2.7.4 Conservative Management

A non‐surgical treatment approach for burn patients encompassing fluid administration, wound care, antibiotic therapy, analgesic administration, fluid resuscitation, and tetanus toxoid prophylaxis, administered individually or in combination [31].

#### 2.7.5 Pre‐Hospital Intervention

Any treatment, substance, or wound care measure, whether clinically appropriate or not, applied to the burn wound before the patient's arrival at a formal health facility, by the patient, a family member, bystander, or community health worker. This includes traditional remedies (butter, oil, egg white, and herbal preparations), pharmaceutical products (topical ointments, and analgesics), physical measures (cold or warm water, wound covering), and other substances (toothpaste, petroleum products, and charcoal) [32, 33].

#### 2.7.6 Outcome of Burn Injury

The health status of participants following hospital care, classified as either discharged with complications or discharged without complications, as documented in the medical chart.

#### 2.7.7 Complications of Burn Injuries

Adverse sequelae resulting from burn or scald injuries, including shock, heat exhaustion, wound infection, and scarring, which may prolong hospitalization and adversely affect recovery.

#### 2.7.8 Discharge With a Complication

Patients discharged with one or more of the following conditions, contracture, disfigurement, amputation, skin graft scarring, or death, as documented in the medical chart, are considered to have experienced a poor outcome following burn injury.

#### 2.7.9 Disfigurement

A permanent or severe alteration of a person's physical appearance resulting from a medical cause, including disease, congenital defect, or injury such as a burn wound [34].

#### 2.7.10 Duration of Presentation

The time elapsed between injury occurrence and initial hospital presentation, classified as early if the patient presented within 24 h of injury, and late if presentation occurred after 24 h.

### 2.8 Data Collection Procedures

A structured data extraction checklist, prepared in English and adapted from previous studies [2, 5, 16, 23, 35], was utilized to ensure consistency and accuracy. Three Bachelor of Science (BSc) Nurses were recruited as data collectors and one MSc Nurse had supervised the entire collection process. The process involved reviewing patient charts to extract relevant information systematically. Missing or ambiguous information in patient charts was managed using single imputation to minimize data loss and maintain sample size.

### 2.9 Data Quality Assurance

To ensure data quality, a 2‐days training session was conducted for data collectors and supervisors prior to data collection. The training covered data collection techniques, ethical considerations during chart reviews, and detailed instructions on the use of the data collection tool and the Kobo Collect application. Each item in the tool was thoroughly explained to ensure clarity and consistency among data collectors.

A pre‐test was conducted on 5% of the sample (*n* = 15) at Arba Minch General Hospital to validate the reliability of the data extraction tool. Based on pre‐test findings, necessary corrections and adjustments were made by the principal investigator and supervisors before initiating the actual data collection. Completed checklists were reviewed daily for completeness and consistency prior to data entry. Data verification was performed by cross‐checking extracted information against the original patient charts. Double data entry and regular consistency checks were additionally conducted to minimize entry errors and enhance the reliability of the dataset.

Throughout the data collection period, the principal investigator closely supervised the process on a daily basis, ensuring strict adherence to protocols and maintaining data quality standards. Any improperly collected data were identified, rechecked, and clarified through direct communication with the respective data collectors to ensure the accuracy and completeness of the final dataset.

### 2.10 Data Processing and Analysis

Data were collected using Kobo Toolbox version 1.3 and subsequently exported to Statistical Package for Social Sciences (SPSS) version 26 for analysis. Descriptive statistics were used to summarize socio‐demographic, clinical, and treatment‐related variables. Categorical variables are presented as frequencies and proportions, while continuous variables are reported as mean with SD. Normality of continuous variables was assessed using the Shapiro–Wilk test (*p* < 0.05), and multicollinearity among independent variables was examined using the Variance Inflation Factor (VIF), with a maximum VIF of 2.10, indicating no significant multicollinearity.

Binary logistic regression model was used to examine the association between each independent variable and the outcome variable; bivariate analysis was performed to examine the unadjusted association between each independent variable and the outcome variable discharge with complications reported as Crude Odds Ratios (COR) with 95% confidence intervals (CI). Independent variables achieving a *p*‐value ≤ 0.25 in bivariate analysis were subsequently entered into the multivariable analysis to control for potential confounding factors and identify independent predictors of the outcome.

Results of the multivariable analysis are reported as Adjusted Odds Ratios (AOR) with 95% CI and corresponding *p*‐values. The goodness‐of‐fit of the final multivariable model was assessed using the Hosmer‐Lemeshow test, yielding a non‐significant *p*‐value of 0.12, indicating adequate model fit. All analyzes were pre‐specified based on the study objectives; no exploratory or post‐hoc subgroup analyzes were performed. All statistical tests were two‐sided, and statistical significance was declared at *p* < 0.05.

### 2.11 Ethical Considerations

The study was conducted in accordance with local regulations, institutional requirements, and the principles of the Declaration of Helsinki [36]. Ethical approval for the study was obtained from College of Health Sciences and Medicine of Wolaita Sodo University Institutional Research Ethics Review Board, with certificate reference number: CHSM/ERC/59/17. Based on this approval, an official letter was issued by the Department of Medicine to the administration of Wolaita Sodo University Comprehensive Specialized Hospital (WSUCSH) to request permission for conducting the study.

The objectives and significance of the research were explained to the hospital administration. Subsequently, the hospital manager provided a formal letter of cooperation to the relevant units, facilitating access to patient records. Data collection was conducted exclusively from patient charts, ensuring that no direct interaction with patients occurred.

To maintain confidentiality and protect the privacy of participants, code numbers were assigned to all data throughout the study, and patient names were excluded from the data collection and analysis process. Since the study was a retrospective chart review and informed consent was waived by the Institutional Review Board. Additionally, all collected data were securely stored and used solely for the purposes of this research, adhering to strict ethical and professional standards.

## 3 Results

### 3.1 Socio‐Demographic Characteristics of Participants

The study analyzed 271 burn injury cases out of a total sample size of 286, yielding a response rate of 94.8%. Fifteen cards were excluded due to incomplete or missing critical information relevant to the study. The mean age of participants was 14.0 years (SD = 13.8). The majority of the participants (64.2%) were below 15 years of age, while 32.1% fell within the 16–60 years age group, and only 3.7% were aged 61 years or above. Regarding sex, males accounted for 51.7% of the participants, while females made up 48.3%. In terms of residence, 52.8% of the participants resided in rural areas, and 47.2% were from urban areas. Marital status was recorded for 94 adult participants, among whom 19.9% were single, 13.7% were married, and 1.1% were widowed.

Marital status data is only available for 94 participants due to the exclusion of pediatric cases and missing registration information in the patient records (See Table 2 for more information).

**Table 2:** Socio‐demographic characteristics of participants at the University Comprehensive Specialized Hospital, Wolaita Zone, South Ethiopia, 2024 (*n* = 271).

Characteristics	Categories	Frequency (*n* = 271)	Percent (%)
Age	< 15 Years	174	64.2
	16–60Years	87	32.1
	≥ 61 Years	10	3.7
Sex	Male	140	51.7
	Female	131	48.3
Residence	Urban	128	47.2
	Rural	143	52.8
Marital status (*n* = 94)	Single	54	19.9
	Married	37	13.7
	Window	3	1.1

### 3.2 Clinical‐Related Characteristics of Participants

The majority of burn injuries among the participants were classified as second‐degree superficial burns, accounting for nearly 65% of the cases. This was followed by second‐degree deep burns, observed in about 24%, while third‐degree burns, also known as full‐thickness burns, were less common at 7%. First‐degree burns were the least reported type, occurring in only 4% of the cases.

In terms of the extent of burn injuries, close to 45% of the participants had burns covering less than 10% of their TBSA. Burns involving 10%–20% of the body were reported in nearly 29%, while a smaller proportion, about 14%, had burns covering between 20%–30%. Only 12% experienced severe burns that affected more than 30% of their body surface area.

When considering the location of burn injuries, the upper extremities were the most commonly affected body parts, accounting for 38% of cases, followed by the lower extremities at 25%. Injuries involving the anterior trunk were observed in 14% of participants, while burns on the posterior trunk and head and neck regions accounted for 9% and 12%, respectively. The perineal area was the least affected at 2%.

Scald burns emerged as the leading cause of injury, accounting for 62% of the cases, while flame burns were the second most common cause at 21%. Electrical burns contributed to 9%, and contact burns were the least frequent at 8%. Among electrical burns, high‐voltage injuries were far more prevalent, making up 8%, compared to low‐voltage incidents, which were rare at less than 1%.

The majority of participants, approximately 65%, sought medical attention within the first 24 h of the injury, categorized as early presentation. However, a significant portion, about 35%, presented late, arriving at the hospital more than 24 h after the burn occurred.

**Figure 1:**
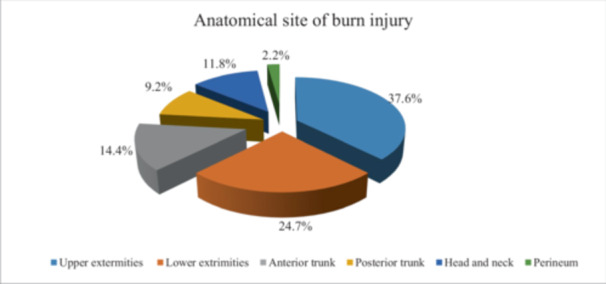
Schematic presentation on anatomical site of burn injury among hospitalized patients at the University Comprehensive Specialized Hospital, Wolaita Zone, South Ethiopia, 2024 (*n* = 271).

**Figure 2:**
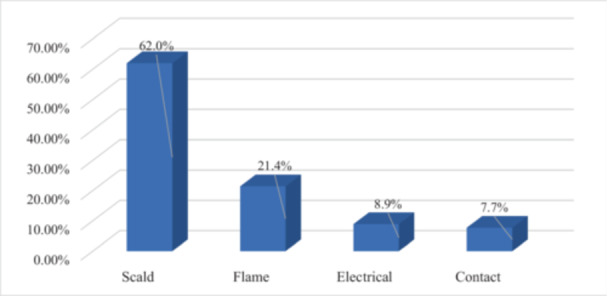
Schematic presentation of cause of burn among burn patients attending at the University Comprehensive Specialized Hospital, Wolaita Zone, South Ethiopia, 2024 (*n* = 271).

**Figure 3:**
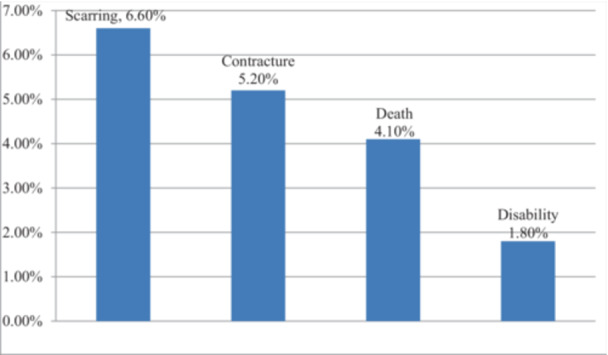
Schematic presentation of the types of complications among burn injury patients hospitalized at the University Comprehensive Specialized Hospital, Wolaita Zone, Southern Ethiopia, 2024 (*n* = 271).

All the burn injuries were reported as accidental, with no cases of intentional or assault‐related burns. The home environment was identified as the primary location where burn injuries occurred, affecting 89% of participants. Burns occurring at workplaces were far less common at 8%, while only a small proportion, about 3%, took place on the street (See Table 3, Figures 1, 2, 3 for more information).

**Table 3:** Clinical‐related characteristics of participants at the University Comprehensive Specialized Hospital, Wolaita Zone, South Ethiopia, 2024 (*n* = 271).

Characteristics	Categories	Frequency (*n* = 271)	Percent (%)
Depth of burn injury	First degree	12	4.4
	Second degree superficial	175	64.6
	Second degree deep	64	23.6
	Third degree/full sickness	20	7.4
The extent of burn injury	< 10%	121	44.6
	10%–20%	78	28.8
	21%–30%	38	14.0
	≥ 30%	34	12.5
Anatomic location of burn injuries	Upper extremities	102	37.6
	Lower extremities	67	24.7
	Anterior trunk	39	14.4
	Posterior trunk	25	9.2
	Head and neck	32	11.8
	Perineum	6	2.2
Cause of burn	Scald	168	62.0
	Flame	58	21.4
	Electrical	24	8.9
	Contact	21	7.7
If Electrical which electrical burn (*n* = 24)	High Voltage	22	8.1
	Low Voltage	2	0.7
Duration of presentation	< 24 h (Early)	175	64.6
	≥ 24 h (Late)	96	35.4
The main circumstance of surrounding burn	Accident	271	100
	Assault	0	0.0
Place of burn occurrence	Home	241	88.9
	Street	8	3.0
	Workplace	22	8.1

### 3.3 Health Service‐Related Characteristics of Participants

Among the participants, only 28.4% received pre‐hospital interventions, while 71.6% had no form of care prior to hospital arrival.

The length of hospital stay varied, with 78% of patients hospitalized for ≤ 15 days, 8.5% for 16–25 days, and 13.3% for ≥ 26 days. The mean duration of hospitalization was 11.63 ± 8.0 days.

Regarding treatment, antibiotics were administered to 71.2% of patients, while 28.8% did not receive them. Pain management was provided to 76% of participants, whereas 24% received no analgesic intervention. Fluid and electrolyte therapy was given to 77.1% of patients, while 22.9% did not receive such support (See Table 4 for more information).

**Table 4:** Health service‐related characteristics of participants at the University Comprehensive Specialized Hospital, Wolaita Zone, South Ethiopia, 2024 (*n* = 271).

Characteristics	Categories	Frequency (*n* = 271)	Percent (%)
Pre‐hospital intervention	Yes	77	28.4
	No	194	71.6
Length of hospital stays	≤ 15 days	212	78.2
	16–25 days	23	8.5
	≥ 26 days	36	13.3
Used antibiotic	Yes	193	71.2
	No	78	28.8
Used pain management	Yes	206	76.0
	No	65	24.0
Used fluid and electrolyte	Yes	209	77.1
	No	62	22.9

### 3.4 Health‐Related Factors

Among the participants, 14% had pre‐existing co‐morbidities, while the majority, 86%, reported no underlying health conditions. Of those with co‐morbidities, 8.1% had Epilepsy, 1.1% had Diabetes Mellitus, and 2.2% had Psychiatric problems, while 2.6% reported other conditions (See Table 5 for more information).

**Table 5:** Health‐related factors of burn injury at the University Comprehensive Specialized Hospital, Wolaita Zone, South Ethiopia, 2024 (*n* = 271).

Characteristics	Categories	Frequency (*n* = 271)	Percent (%)
Co‐morbidity	Yes	38	14.0
	No	233	86.0
Type of co‐morbid diseases (*n* = 38)	Epileptic	22	8.1
	DM	3	1.1
	Psychiatric problem	6	2.2
	Others	7	2.6

### 3.5 Management and Outcomes of Burn Injuries in the Burn Ward

Regarding the outcome of burn injuries, 82.3% of participants were released without complications, and 17.7% were treated with complications. Among those released with complications, 4.1% died, 1.8% developed disabilities, 6.6% had scarring and 5.2% had contractures.

The most common procedure, carried out in 15.5% of the cases, was wound debridement. Among those who died from burn related complications, sepsis due to the focus of the wound was the most common cause in 1.5% of patients, fasciotomy in 2.2% and contracture release in 0.7%. Shock, including hypovolaemia or sepsis, accounted for 0.7%, while Hospital Acquired Pneumonia (HAP) accounted for 1.1% of the deaths. Other causes, such as sepsis due to gastrointestinal or genitourinary causes, accounted for 0.7% of the total number of cases (See Table 6 for more information).

**Table 6:** Management outcome of burn injury at the University Comprehensive Specialized Hospital, Wolaita Zone, South Ethiopia, 2024 (*n* = 271).

Characteristics	Categories	Frequency	Percent (%)
Outcome of burn injury	Discharged with complication	48	17.7
	Discharged without complication	223	82.3
Surgical intervention done for burn	Wound debridement	42	15.5
	Skin graft	4	1.5
	Fasciotomy	6	2.2
	Contracture release	2	0.7
Outcome with complications (*n* = 48)	Death	11	4.1
	Disability	5	1.8
	Scaring	18	6.6
	Contracture	14	5.2
Reason for death (*n* = 11)	Sepsis of wound focus	4	1.5
	Shock (hypovolemic/septic)	2	0.7
	HAP	3	1.1
	Others	2	0.7

### 3.6 Factors Associated With Treatment Outcomes of Burn Injury

Bi‐variable and multivariable analyzes were conducted to identify risk factors for participants with discharged with complication after burn injury. In bivariable logistic regression analysis, several factors showed an association with discharge with complication, including age, pre‐hospital intervention, cause of burn, fluid and electrolyte replacement, surface area of burn, and length of hospital stay. These variables were included in the multivariable analysis to control for potential confounders.

After adjusting for confounders in the multivariable analysis, four variables were found to have a statistically significant association for discharge with complication. These were age ≥ 60 years (AOR = 4.40; 95% CI: 1.01**–**19.11; *p* = 0.048), having pre‐hospital intervention (AOR = 2.40; 95% CI: 1.15**–**4.98; *p* = 0.019), lack of fluid and electrolyte replacement (AOR = 3.05; 95% CI: 1.39**–**6.70; *p* = 0.005), and BSA ≥ 30% (AOR = 5.20; 95% CI: 1.53**–**17.64; *p* = 0.008).

In this study participants aged 60 years and older were over four times more likely to be discharged with complications compared to those younger than 15 years. Similarly, those who received pre‐hospital intervention were over 2.3 times more likely to have complications compared to those with no pre‐hospital care.

Other factors, like participants who did not receive fluid and electrolyte replacement had a threefold higher risk of complications compared to those who received the intervention. Additionally, patients with burn injuries covering 30% or more of their TBSA were over five times more likely to experience complications than those with burns covering less than 10% (See Table 7 for more information).

**Table 7:** Bivariate and multivariable analysis for management outcome of burn injury in the University Comprehensive Specialized Hospital, Wolaita Zone, South Ethiopia, 2024 (*n* = 271).

Variables	Category	Burn outcome		COR (95% CI)	AOR (95% CI)	*p* value
		Discharged with complication *N* (%)	Discharged without complication *N* (%)			
Age	< 15 years	28 (58.3)	146 (65.5)	1	1	
	16–60 years	16 (36.3)	71 (31.8)	1.17 (0.59**–**2.31)	1.49 (0.68**–**3.29)	0.319
	≥ 60 years	4 (8.3)	6 (2.7)	3.48 (0.92**–**13.12)	4.40 (1.01**–**19.11)	0.048
Pre‐hospital intervention	Done	22 (45.8)	55 (24.7)	2.58 (1.36**–**4.92)	2.40 (1.15**–**4.98)	0.019
	Not done	26 (54.2)	168 (75.3)	1	1	
Cause of Burn	Scald	36 (75.0)	132 (59.2)	2.59 (0.58**–**11.64)	2.92 (0.55**–**15.58)	0.210
	Flame	6 (12.5)	52 (23.3)	1.10 (0.20**–**5.91)	1.32 (0.20,8.54)	0.77
	Electrical	4 (8.3)	20 (9.0)	1.90 (0.31**–**11.61)	2.17 (0.28**–**16.57)	0.455
	Contact	2 (4.2)	19 (8.5)	1	1	
Fluid and electrolyte replacement	Yes	30 (62.5)	179 (80.3)	1	1	
	No	18 (37.5)	44 (19.7)	2.44 (1.25**–**4.77)	3.05 (1.39**–**6.70)	0.005
Surface area	< 10%	13 (27.1)	108 (48.4)	1	1	
	10%–20%	12 (25.0)	66 (29.6)	1.51 (0.65**–**3.51)	1.92 (0.74**–**4.98)	0.181
	21%–30%	8 (16.7)	30 (13.5)	2.21 (0.84**–**5.84)	1.52 (0.41,**–**5.61)	0.527
	≥ 30%	15 (31.3)	19 (8.5)	6.56 (2.70**–**15.95)	5.20 (1.53**–**17.64)	0.008
Length of hospital Stays	≤ 15 days	28 (58.3)	184 (82.5)	1	1	
	16–25 days	6 (12.5)	17 (7.6)	2.32 (0.84**–**6.38)	2.42 (0.70**–**8.42)	0.163
	≥ 16 days	14 (29.2)	22 (9.9)	4.18 (1.91**–**9.11)	2.47 (0.78**–**7.82)	0.124

## 4 Discussion

In this study, 17.7% (95% CI: 13.0%–22.0%) of burn injury patients were discharged with complications. Significant factors associated with discharge with complications included receiving pre‐hospital intervention, absence of fluid and electrolyte replacement, burns involving ≥ 30% of TBSA, and age ≥ 60 years.

In this study, the finding that 17.7% of burn injury patients were discharged with complications aligns with reports from other institutions in Ethiopia, such as Ayder Referral Hospital, which documented a complication rate of 17.3%, and at Addis Ababa Burn Emergency and Trauma Hospital found that only 14.2% of patients were discharged with complications [23, 35] and additionally, a study conducted Ghana was (21.3%) [37]. The similarities in these findings may be attributed to common factors influencing burn outcomes across these healthcare settings, such as the prevalence of scald burns, which are particularly common in pediatric populations, and the overall healthcare infrastructure available for managing burn injuries.

In this study, 82.3% of participants were discharged without complications, a significantly higher proportion than reported in South West Ethiopia [38] (65.1%) and North Showa Zone (59.1%) [39], likely due to differences in sample size, methodology, and healthcare quality. However, the rate of complications, including a mortality rate of 4.1%, exceeded those reported in Nigeria (3.8%) [40], Egypt (2.5%) [41], Iraq (2.7%) [42], the Netherlands (3.2%) [43], Turkey (3.8%) [34], and Pakistan (2.35%) [44]. This disparity could be attributed to the lack of specialized burn units, insufficiently trained personnel, and limited infrastructure in the study setting, unlike the advanced facilities available in the compared regions. Delayed access to care, inadequate pre‐hospital services, and insufficient resources for infection control and rehabilitation further compounded these outcomes, emphasizing the need for investment in dedicated burn care services to improve patient outcomes and align with global standards.

The odds of developing discharge with complications for patients aged ≥ 60 years are four times higher compared to those aged < 15 years. This finding is supported by studies conducted in Addis Ababa [23], New york [45], Netherland [46], and Portugal [46], which found that older burn patients were more likely to experience poor outcomes, including prolonged hospital stays and increased complications. The possible explanation for this might be the age‐related physiological changes, such as thinner skin, reduced immune function, and slower wound healing. Therefore, elderly burn patients require more intensive monitoring and care to prevent and manage complications effectively [47].

The odds of developing complications were 2.4 times higher among patients who received pre‐hospital interventions compared to those who did not. This finding is consistent with studies conducted in South Western and North Shewa, Ethiopia [38, 39], and evidence from Ethiopia and the broader African context, where pre‐hospital burn care is largely characterized by the application of harmful traditional remedies, including butter, oil, egg white, toothpaste, and petroleum products, rather than evidence‐based first aid, such practices introduce infection, trap wound heat, delay definitive care, and worsen clinical outcomes. These interventions may exacerbate burn severity or delay proper treatment [33, 39]. Additionally, inadequate pre‐hospital care systems in these regions could contribute to poor outcomes. However, this result contrasts with findings from studies conducted in Tigray and Tanzania [35, 48]. The disparity may stem from differences in pre‐existing medical conditions, the extent and depth of burn injuries, and variations in cultural practices related to burn management. In stark contrast, pre‐hospital burn management in developed countries involves structured emergency medical systems delivering evidence‐based interventions prior to hospital arrival. In Germany and Switzerland, pre‐hospital care for burn patients includes opioid‐based analgesia, crystalloid fluid resuscitation, accurate burn size estimation, and timely transport to specialized burn centers [49, 50]. Across the United Kingdom, Europe, and Australia, the application of 20 min of cool running water within 3 h of injury is standard practice, supported by national guidelines and robust evidence demonstrating a 48% reduction in ICU admission and significant reduction in hospital length of stay [51, 52]. This stark disparity underscores the urgent need for community‐level burn first aid education and strengthened pre‐hospital emergency systems in Ethiopia and similar resource‐limited settings [53].

The odds of developing complications upon discharge are three times higher for burn patients who did not receive adequate fluid and electrolyte replacement compared to those who received proper fluid management. This result supported with findings from North Shewa and Tigray region of Ethiopia [35, 39] which demonstrated that failure to provide appropriate fluid resuscitation within the first 24 h of burn injury significantly increases the risk of complications such as multi‐organ failure and death. The possible explanation for this association is that inadequate fluid replacement can lead to hypovolemic shock, kidney dysfunction, and impaired tissue perfusion, all of which can delay recovery and contribute to adverse outcomes [48]. Therefore, timely and appropriate fluid management is critical to preventing these complications.

Burn patients with a BSA ≥ 30% are 5.2 times more likely to develop complications upon discharge compared to those with a BSA < 30%. This finding is supported by study done in Bahir Dar, South Gondar and South West Ethiopia and [31, 38, 54], that found that patients with extensive burns, covering ≥ 30% of their body, had significantly higher rates of complications, including infections, sepsis, and organ dysfunction. The possible explanation for this might be that larger burn areas result in more severe systemic effects, including significant fluid loss, electrolyte imbalances, and increased risk of infection. The body's ability to recover is compromised when a large portion of skin is damaged, which is essential for fluid and temperature regulation. Therefore, these patients require more intensive and prolonged care to manage the complications associated with severe burns [55].

## 5 Limitation of the Study

This study has several limitations that should be acknowledged. The cross‐sectional design does not allow for establishing temporal or causal relationships between the identified factors and burn outcomes. Data were collected from medical records, which may be incomplete or inconsistently documented, potentially affecting the accuracy of information on pre‐hospital interventions and fluid management. The study was conducted at a single tertiary hospital, which may limit the generalizability of findings to other facilities or regions with different patient populations and resources. Some potentially influential variables, including nutritional status, comorbidities, socioeconomic factors, and time from injury to admission, were not systematically captured, which could result in residual confounding. Although consecutive sampling included all eligible patients, there remains a possibility of selection bias if patients with extreme outcomes were more likely to be admitted or documented. Conducting multi‐center, prospective studies incorporating these additional variables would provide a more comprehensive understanding of burn outcomes in similar resource‐limited settings.

## 6 Conclusion

Advanced age (≥ 60 years), inappropriate pre‐hospital interventions, insufficient fluid and electrolyte replacement, and extensive burns (≥ 30% TBSA) emerged as key independent determinants of discharge with complications. These findings underscore the urgent need for targeted, context‐specific strategies: age‐tailored clinical management for elderly patients, community‐level burn first aid education to discourage harmful pre‐hospital practices, timely and adequate fluid resuscitation in adherence to established protocols, and prioritized intensive care for patients with extensive burns. Addressing these modifiable determinants offers a practical pathway for health systems in resource‐limited settings to reduce burn‐related complications, inform evidence‐based policy, and ultimately improve patient outcomes in South Ethiopia and similar settings.

## 7 Recommendations

The study identified advanced age (≥ 60 years), inappropriate pre‐hospital interventions, inadequate fluid and electrolyte replacement, and extensive burns (≥ 30% TBSA) as predictors of discharge with complications. For patients, these findings highlight the importance of timely and appropriate care, particularly for the elderly and those with severe burns. Healthcare professionals and hospital teams can use these results to strengthen monitoring, optimize fluid management, and prioritize high‐risk patients. Researchers may build on this evidence to investigate targeted interventions or age‐specific strategies in similar settings. At the policy level, the findings support the development of context‐appropriate burn management guidelines, investment in training for pre‐hospital responders, and resource allocation to ensure effective burn care, ultimately aiming to reduce complications and improve outcomes across hospitals in resource‐limited settings.

## Author Contributions

**Sewnet Ejigu:** conceptualization, investigation, validation, formal analysis, writing – original draft, resources, project administration. **Tesfaw Alemu Legas:** methodology, software, data curation, formal analysis, writing – original draft. **Samson Kastro Dake:** methodology, validation, supervision, visualization. **Natnael Sima:** methodology, data curation, supervision, visualization, validation. **Samuel Minalkew:** methodology, data curation, software, visualization, writing – original draft. **Mulugeta Edao Shate:** writing – review and editing, methodology, data curation, validation. **Wondimagegn Genaneh Shiferaw:** methodology, software, validation, visualization, writing – review and editing, data curation.

## AI Use Declaration

During the preparation of this manuscript, the authors used Claude (Anthropic) exclusively for language editing and academic tone refinement. All intellectual content, data, analyses, interpretations, and conclusions are entirely the work of the authors. The authors take full responsibility for the accuracy, integrity, and originality of all content presented in this manuscript.

## Funding

The authors have nothing to report.

## Ethics Statement

The study was conducted in accordance with local regulations, institutional requirements, and the principles of the Declaration of Helsinki [56]. Ethical approval was obtained from the Institutional Review Board (IRB) of Wolaita Sodo University, College of Health Science and Medicine (Ref: CHSM/ERC/59/17). As the study employed a retrospective chart review design with no direct patient contact, informed consent was waived by the IRB. Patient confidentiality was strictly maintained, and all data were anonymized prior to analysis.

## Consent

Ethical approval was obtained from the Ethical Review Committee of Wolaita Sodo University, College of Health Science and Medicine. This study was a retrospective chart review, and informed consent was waived by the Wolaita Sodo University, College of Health Science and Medicine, Ethical review Committee.

## Conflicts of Interest

The authors declare no conflicts of interest.

## Transparency Statement

Dr. Sewnet Ejigu (MD), as the manuscript guarantor, affirms that this manuscript is an honest, accurate, and transparent account of the study being reported; that no important aspects of the study have been omitted; and that any discrepancies from the study as planned have been explained.

## Data Availability

The data that support the findings of this study are available on request from the corresponding author. The data are not publicly available due to privacy or ethical restrictions. This study was based on data extracted directly from patient medical charts at Wolaita Sodo University Comprehensive Specialized Hospital. The derived data supporting the findings of this study are not publicly available due to patient confidentiality constraints but are available from the Principal Investigator, Dr. Sewnet Ejigu (MD), Department of Medicine, Wolaita Sodo University School of Medicine (email: Sewnet4ejigu3@gmail.com), upon reasonable request.
